# Older working adults in the HEAF study are more likely to report loneliness after two years of follow-up if they have negative perceptions of their work quality

**DOI:** 10.1186/s12889-021-10610-5

**Published:** 2021-03-23

**Authors:** Gregorio Bevilacqua, Stefania D’Angelo, Georgia Ntani, Holly Emma Syddall, Elizabeth Clare Harris, Cathy Linaker, Martin Stevens, Cyrus Cooper, Karen Walker-Bone

**Affiliations:** 1 MRC Lifecourse Epidemiology Unit, University of Southampton, Southampton General Hospital, Southampton, SO16 6YD UK; 2 MRC Versus Arthritis Centre for Musculoskeletal Health and Work, MRC Lifecourse Epidemiology Unit, University of Southampton, Southampton, England

**Keywords:** Perceived work quality, Psychosocial work factors, Loneliness, Retirement, Older workers

## Abstract

**Background:**

Loneliness is an important public health issue associated with mortality and morbidity. Often researched amongst older people, less is known about risk factors for loneliness among adults aged 50–64 years who are in work. We investigated (a) if exit from the workforce increases the odds of loneliness; (b) whether adverse psychosocial work factors are associated with increased odds of loneliness over 2 years of follow-up; and (c) whether the association is stronger among subjects still working compared with those who have exited the workforce.

**Methods:**

Data came from the Health and Employment After Fifty (HEAF) study, a large population cohort who provided questionnaire information about work and health at baseline and 2 annual follow-ups. Logistic regression was used to explore the association between psychosocial risk factors and loneliness at follow-up 2, with adjustment for loneliness at baseline, sex, age, self-rated health, living alone, and mental health diagnosis.

**Results:**

Of the initial 8134 participants, 4521 were working at baseline and provided data for this analysis. Of those, 507 (11.2%) were defined as lonely at 2 years’ follow-up. Exiting the workforce was not significantly associated with loneliness (OR = 1.1, 95%CI: 0.7–1.7). However, negative psychosocial work factors predicted loneliness at follow-up. After mutual adjustment, lack of choice at work (OR: 1.5, 95%CI: 1.1–1.9), often lying awake worrying about work (OR: 1.4, 95%CI: 1.0–1.9) and perceived not coping with physical demands of the job (OR: 1.3, 95%CI: 1.0–1.7) were independent predictors, with associations robust to adjustment for demographic factors and health. Associations were only slightly altered when we restricted the sample to those who remained in work until the end of follow-up.

**Conclusions:**

Loneliness amongst middle-aged working adults is not predicted by permanent work exit but is predicted by individuals’ perceptions about their work. Provision of good-quality work, matched to the capacity of the older worker, could prevent loneliness.

**Supplementary Information:**

The online version contains supplementary material available at 10.1186/s12889-021-10610-5.

## Background

Loneliness is an important public health issue. Defined as a subjective and negative evaluation of the gap between an individual’s desired and actual quantity and quality of social relationships [1, 2], it has been consistently linked with higher rates of mortality and worse cardiovascular and mental health outcomes [3]. In fact, the size and burden of the health risks associated with loneliness have been reported to be similar in magnitude to those associated with other major health risk factors such as obesity and smoking [4]. Consequently, there have been calls for policy makers and health commissioners to consider loneliness as an important risk factor for mortality and morbidity and to develop prevention strategies [3]. To achieve this, good quality evidence about the important risk factors for loneliness is required.

As life expectancy is increasing, population demographics are changing rapidly with a steady rise in the proportion of economically inactive adults, and an attendant financial concern of funding pensions for all. Thus, there is a growing need to encourage people to stay in work to older ages. Working is generally thought to be good for health, providing a sense of purpose and belonging [5], while at the same time offering the opportunity to participate in social interactions [6]. Indeed, studies from Australia and Croatia have shown unemployment to be associated with loneliness [7, 8]. As such, one might hypothesize that any exit from the workforce, either through healthy retirement or unemployment, increases the risk of loneliness. However, much of the loneliness literature has focussed on older people, amongst whom retirement from the working environment is thought to have played a role because of severance of work-associated social relationships, making it difficult to test this hypothesis [9–25]. Were this hypothesis true, if more people remain in work to older ages, we might expect a beneficial effect on the health of older people by reducing the risk of loneliness.

Psychosocial work characteristics can be defined as: interactions between and among work environment, job content, organisational conditions and workers’ capacities, needs, culture, personal extra-job considerations that may, through perceptions and experience, influence health, work performance and job satisfaction [26]. Previous studies have reported that adverse psychosocial work factors are associated with a high risk of subsequent depressive symptoms or episodes [27]. Little is currently known about the prevalence of, and risk factors for, loneliness amongst adults aged 50–64 years who are, in many cases, still in paid work or moving towards a planned retirement. Not all jobs are the same, and it is indeed possible that poor-quality jobs (e.g. those with poorer psychosocial environments) may not offer much protection against loneliness. One longitudinal study conducted amongst a Chinese cohort of older adults found that the risk of future loneliness was affected by the types of occupation in which individuals were employed (in particular, participants with office-based jobs showed reduced risk of incident loneliness). A further small study including a younger group of workers (mean age 27.6 ± 2.25 years) in Turkey reported that job dissatisfaction was associated with greater risk of loneliness [28, 29]. Hypothesizing that adverse psychosocial work factors indeed impact the risk of loneliness, one might expect their effects to be stronger among those still in work than among people who may have experienced an adverse working environment in the past but have now exited the workforce.

Therefore, we used data from the Health and Employment After Fifty (HEAF) cohort to assess the effect of exiting the workforce on subsequent development of loneliness and to investigate the risk factors for loneliness over 2 years of follow-up amongst older workers (aged 50–64 years at baseline), with a particular focus on psychosocial work factors. We also tested the hypothesis that the effect would be more pronounced among older workers still in work than those who have exited the workforce.

## Methods

Analyses were based on data collected at baseline and at 2 further annual follow-ups from the HEAF cohort study, the detailed design and methods of which have been reported elsewhere [30]. Briefly, the sampling frame for this cohort study were 24 English General Practices geographically spread all across England and representing every decile of social deprivation according to the 2010 English Index of Multiple Deprivation [31]. General practice registers are used frequently for population sampling because the vast majority of the British population is registered with a general practice for the provision of primary health care in the publically-funded UK National Health System (NHS). Questionnaires were posted over an 18-month period commencing January 2013 to all men and women aged 50–64 years registered with each practice. Participants were asked to return a baseline questionnaire to the study team via post, as well as written consent to receive annual follow-up questionnaires and to linkage of their questionnaire responses with their CPRD record.

The baseline questionnaire collected information about: demographic factors; lifestyle; work characteristics; self-rated health (SRH); depression (assessed using the Centre for Epidemiological Studies instrument (CES-D)); financial circumstances; and retirement intentions. The follow-up questionnaires, which were mailed after approximately 12- and 24-months, enquired whether they had exited work since the previous questionnaire. Participants who were working at baseline, reported leaving a job at one-year follow-up, and remained out of work at 2 years’ follow-up were defined as having made a permanent job exit for these analyses. The CES-D instrument was included in every questionnaire.

### Outcome: loneliness at 2-year follow-up

Loneliness was assessed at each time point by a single question from the CES-D scale: “how often in the past 7 days have you felt lonely?” Possible answers were “rarely or none of the time”, “some or a little of the time”, “occasionally or a moderate amount of the time”, “most or all of the time”. This single-item question has been previously shown to correlate moderately well with findings obtained from the widely used UCLA three-item loneliness scale (*r* = 0.54, *p* < 0.01) [32]. Respondents were defined as reporting loneliness if they reported feeling lonely “occasionally or a moderate amount” or “most or all the time”, but not if they gave any other response.

### Independent variables: psychosocial work characteristics

In the baseline questionnaire, everybody in paid employment was asked to complete questions about the psychosocial characteristics of their current job, including whether they perceived: they had choice about how they worked; they received support from colleagues or managers; that they were appreciated, that they felt they achieved (all coded as rarely/never vs often/sometimes). They were also asked: whether they often lay awake worrying about work (often vs sometimes/rarely); how satisfied they felt (dissatisfied vs satisfied); and how well they felt they were able to cope with the physical and mental demands of the job (at least some difficulty vs easily). These questions were designed to ascertain the individual’s perceptions and feelings about their job, and are compatible with previously developed models such as the effort–reward imbalance model [33] and the Copenhagen Psychosocial Questionnaire [34], which focus on comparable domains such as perceived demands and rewards at work, interpersonal relations at work, and job security. These responses assessed at baseline were evaluated as predictors of subsequent loneliness.

### Potential confounding factors

The baseline questionnaire provided information about: age; sex; highest educational qualification; home ownership (coded as rented/rent free vs mortgaged/owned outright); number of people living in the household (coded as living alone vs not), and self-rated health (SRH) (fair/poor vs at least good). We also extracted information from participants’ CPRD records relating to a diagnosis of, and/or treatment for, a common mental health condition in the past 12 months.

### Statistical analysis

The analyses focused on loneliness reported at 2 years of follow-up, with adjustment for loneliness at baseline. Participants’ characteristics were described using frequency and percentage distributions, means and standard deviations, depending of the nature of the variable. Differences between those who reported being lonely and those who did not at 2 years of follow-up were assessed using Chi-square test for categorical variables, and *t*-test for continuous variables.

Given the complexity of the relationships between socio-economic characteristics and psychosocial work factors, potential confounding variables in the association between work factors and loneliness were identified ahead of the analysis using a Directed Acyclic Graph (DAG), with the software DAGitty [35]. DAGs are graphical representations of casual effects between variables, which are created from the pre-existing evidence about the causal relations for the effects studied. DAGs provide an efficient and objective means of selecting confounders which are then adjusted for in the statistical analysis [36]. In brief, firstly we selected variables to be considered for inclusion based on our literature review, then we took each variable in turn, evaluating any possible association between it and the other considered variables based on the published evidence and plausibility of associations. Considered for inclusion were: age, sex, educational attainment, home ownership, living alone, self-rated health and diagnosis of, or treatment for, a common mental health condition in the preceding 12 months from the CPRD record. After completion of the DAG (Additional file 1), the following factors remained as confounders: age, sex, living alone, SRH, and mental health diagnosis.

Logistic regression modelling was used to explore the association between baseline psychosocial work factors and loneliness at 2 years of follow-up after adjusting for the pre-defined confounders. Estimates of effect were expressed as odds ratios (ORs) and 95% Confidence Intervals (95% CI). All models were also additionally adjusted for loneliness at baseline. Firstly, each of the psychosocial work factors was adjusted for the confounders in separate regression models. Lastly, all psychosocial work factors significant at the 5% level in the previous analysis stage were mutually adjusted.

We subsequently explored whether the associations between psychosocial aspects of work were more pronounced among participants still working throughout the duration of follow-up by refitting the final mutually adjusted model by work status at the end of follow-up.

The main analyses were conducted for men and women collectively. Final models were also fitted separately for men and women given that men and women have different types of jobs and profiles of health conditions. All analyses were carried out with Stata software v15.1.

## Results

A total of 8134 people returned a baseline questionnaire and gave their consent to be followed-up. Of these, 5473 were working at the time of the baseline questionnaire, while the rest had stopped working before baseline. Among workers at baseline, the prevalence of all adverse psychosocial work factors was higher among those who reported loneliness at baseline, when compared with those who did not (Additional file 2). For instance, not being able to cope with the mental demands of the job was reported by 29% of people without loneliness as compared with 53% of those reporting loneliness.

Amongst workers at baseline, 4559 (83%) returned a usable questionnaire at 2 years of follow-up, amongst whom 4521 completed the question about loneliness. The demographic, socio-economic, lifestyle, health and work characteristics of the 4559 who responded to the 2-year follow-up questionnaire were not significantly different from the characteristics of all participants in paid work at baseline and not successfully followed-up (data not shown).

Amongst those successfully followed-up (2148 men and 2373 women), 507 (11.2%) fulfilled our case definition for loneliness at 2-years, with slightly higher prevalence amongst women (12.8%) than men (9.5%). In total, 318 (7.0%) participants had permanently left their job during the 2-year follow-up period (89% of those who exited reported themselves as retired while the remaining 11% reported that they had become unemployed). The crude prevalence of loneliness was slightly higher among those who permanently exited from work as compared with those still working (13.5% vs 11%), but, after adjustment for age, sex, SRH, living alone, baseline loneliness, and mental health diagnosis we found that those who exited the workforce were not at significantly increased odds of loneliness (OR = 1.1, 95%CI: 0.7–1.7).

Table 1 shows the baseline characteristics of participants by their response about loneliness at 2-year follow-up. When compared with participants who reported rarely or never feeling lonely, those who experienced loneliness at 2-year follow-up were more likely to: be women (60% vs 52%); live alone (36% vs 18%); rent rather than own their home (22% vs 10%); have somewhat higher BMI (mean (SD) 27.9 kg/m^2^ (5.9) vs 27.0 kg/m^2^ (4.8)); be current cigarette smokers (15% vs 9%); report fair/poor SRH (31% vs 15%); and have a CPRD-record of a diagnosis of and/or treatment for a common mental health condition in the 12 months before baseline (26% vs 14%).

**Table 1:** Baseline characteristics of participants by loneliness at 2-year follow-up

	Not lonely at FU2 (*n* = 4014)	Lonely at FU2 (*n* = 507)	*P*-value±
Reporting loneliness at baseline	247 (6.2)	207 (40.8)	< 0.001
Sex, women	2069 (51.5)	304 (60.0)	< 0.001
Age (years) (mean(SD))	57.5 (4.1)	57.1 (4.0)	0.03
Living alone	706 (17.8)	178 (35.5)	< 0.001
Housing tenure, rented/rent free	379 (9.6)	107 (21.6)	< 0.001
Level of education			
No qualification/School	1281 (31.9)	189 (37.3)	0.02
Vocational training certificate	1274 (31.7)	152 (30.0)	
University degree/higher	1459 (36.4)	166 (32.7)	
BMI, mean (SD)	27.0 (4.8)	27.9 (5.9)	< 0.001
Smoking status			
Never	2224 (55.8)	264 (52.7)	0.005
Ex	1387 (34.8)	161 (32.1)	
Current	373 (9.4)	76 (15.2)	
Fair/poor SRH	596 (15.0)	154 (30.9)	< 0.001
CPRD mental health diagnosis	528 (13.7)	124 (25.9)	< 0.001
*Psychosocial work factors*			
Rarely having choice at work	718 (18.2)	153 (30.7)	< 0.001
Lack of support	411 (10.4)	77 (15.4)	0.25
Often lying awake worrying about work	429 (10.8)	127 (25.2)	< 0.001
Rarely feeling achievement	244 (6.2)	61 (12.1)	< 0.001
Rarely feeling appreciated	349 (8.8)	98 (19.5)	< 0.001
Job dissatisfaction	208 (5.3)	76 (15.1)	< 0.001
Not coping with physical demands	1052 (26.5)	223 (44.4)	< 0.001
Not coping with mental demands	1144 (28.9)	237 (47.2)	< 0.001
Permanent job exit	275 (6.9)	43 (8.5)	0.18

Statistics are frequency and percentage distributions within categories of loneliness at 2 years’ follow-up, unless otherwise stated; FU2: 2-year follow-up.

± *P*-value from logistic regression.

Participants with loneliness at 2-years were also more likely to report at baseline that they: often laid awake worrying about work; perceived that they rarely had a choice, rarely felt appreciated or had a feeling of achievement at work; were dissatisfied with their job; and struggled to cope with either mental and/or physical demands of the job when compared with those who did not report loneliness.

Table 2 reports the associations between baseline adverse psychosocial work factors and loneliness at 2-year follow-up, overall and by sex. After adjustment for age, sex, SRH, living alone, baseline loneliness, and mental health diagnosis we found a significantly increased odds of loneliness at 2-year follow-up with most of the adverse psychosocial work factors assessed at baseline. Significant associations were found with perceived lack of choice at work (OR 1.7); often lying awake worrying about work (OR 1.7); job dissatisfaction (OR 1.9); rarely feeling appreciated at work (OR 1.7); not coping with mental demands (OR 1.5) and not coping with physical demands (OR 1.5) at work. When the significant psychosocial work factors were mutually adjusted, perceived lack of choice at work (OR 1.5), and not coping with physical demands at work (OR 1.3) remained associated with increased odds of loneliness at follow-up. The effect estimates provided in Table 2 were similar before and after adjustment for the pre-defined confounders.

**Table 2:** Associations between baseline psychosocial work characteristics and loneliness at follow-up 2, in the overall sample, among men and women, and among participants without job exit 2-year follow-up *

	OR (95%CI)						
	Overall			Men		Women	
Predictor	**All**	**All**	**Participants without job exit**	**All**	**Participants without job exit**	**All**	**Participants without job exit**
	Adjusted for pre-defined confounders^1^	Mutually adjusted^2^	Mutually adjusted^2^	Mutually adjusted^2^	Mutually adjusted^2^	Mutually adjusted^2^	Mutually adjusted^2^
Rarely having choice at work	**1.7 (1.3,2.1)**	**1.5 (1.1,1.9)**	**1.6 (1.2,2.1)**	1.4 (0.9,2.1)	**1.5 (1.0,2.3)**	**1.5 (1.1,2.1)**	**1.7 (1.2,2.3)**
Lack of support	1.3 (0.9,1.8)		–				
Often worrying about work	**1.7 (1.3,2.3)**	**1.4 (1.0,1.9)**	1.4 (0.9,2.0)	1.3 (0.8,2.1)	1.3 (0.8,2.1)	**1.5 (1.0,2.2)**	1.4 (0.9,2.2)
Rarely feeling of achievement	1.2 (0.8,1.7)		–				
Rarely feeling of appreciation	**1.7 (1.2,2.2)**	1.3 (0.9,1.8)	**1.5 (1.0,2.1)**	1.2 (0.7,1.9)	1.2 (0.7,2.0)	1.4 (0.9,2.2)	1.5 (0.9,2.5)
Job dissatisfaction	**1.9 (1.3,2.6)**	1.2 (0.9,1.8)	1.3 (0.8,2.2)	1.2 (0.7,2.1)	1.2 (0.7,2.2)	1.3 (0.8,2.2)	1.2 (0.7,2.1)
Not coping with physical demands	**1.5 (1.2,1.9)**	**1.3 (1.0,1.7)**	1.2 (0.9,1.7)	**1.5 (1.2,2.2)**	1.3 (0.9,2.0)	1.1 (0.8,1.6)	1.1 (0.8,1.6)
Not coping with mental demands	**1.5 (1.2,1.8)**	1.1 (0.9,1.5)	1.2 (0.9,1.6)	1.3 (0.9,2.0)	1.4 (0.9,2.1)	1.0 (0.7,1.5)	1.1 (0.8,1.6)

^1^ Estimates shown come from separate logistic regression models for each of the psychosocial work factors and also adjusted for pre-defined confounders: age, sex, living alone, SRH, baseline loneliness, and mental health diagnosis

^2^ Estimates shown are mutually adjusted, and adjusted for pre-defined confounders: age, sex, living alone, SRH, baseline loneliness, and mental health diagnosis. Sex in not included as adjustment factor in case of stratified analyses

*Bold denotes statistically significant estimates at *p* < 0.05 level of confidence

As we hypothesised that there might be a differential effect of psychosocial work factors on loneliness between participants who had remained in work throughout follow-up and those who had permanently exited the workforce, we re-fitted the final mutually adjusted model by work status at the end of follow-up. As shown in Table 2, significant associations were found between rarely having a choice, not feeling appreciated and subsequent loneliness among participants in employment throughout follow-up. However, effects of psychosocial work factors among participants who had permanently exited their job were weak and not significant (data not shown).

After stratification by sex (Table 2), we found that men who reported that they were not coping with the physical demands of their work were at increased odds of loneliness. However, when we restricted the analyses to men without a job exit, this association did not persist. Among women, in the overall sample, lack of choice and reporting often lying awake worrying about work were significant risk factors for loneliness and lack of choice was still significant when the sample was restricted to women without a job exit.

In a complementary analysis, we explored the risk factors for persistent loneliness (4.6% of the sample) and compared them with those found for new onset loneliness (6.6% of the sample). The results were broadly similar, suggesting that the risk factors for persistent loneliness were not much different from those shown above in relation to incident loneliness over 2 years of follow-up (data not shown).

Finally, in order to test for the potential clustering effect of GPs, we replicated the analyses using random intercept multilevel models. We found a negligible intra-class correlation (approximately zero) so that the resultant effect estimates were almost identical to those presented in Table 2.

## Discussion

In this study we investigated the association between adverse psychosocial work factors and loneliness after 2 years of follow-up amongst 4521 people aged 50–64 years in paid work at baseline. The prevalence of loneliness was 11.2%; this was higher among women than men, and consistent with rates reported in other UK surveys [23, 24, 37–39]. We also found that exiting the workforce was not significantly associated with increased odds of loneliness, while those who reported adverse psychosocial work characteristics at baseline (particularly those reporting lack of choice at work, often lying awake worrying about work, and not coping with physical demands of work) were more likely to report loneliness at 2 years of follow-up. Our results were robust to adjustment for demographic characteristics, health status and loneliness at baseline. In support of our original hypothesis, these findings suggest that poor quality work, as perceived by the individual, is a risk factor for developing loneliness.

Our finding that loneliness was univariately associated with: living alone; renting rather than owning a home; having higher BMI; reporting poor SRH; and having a diagnosis, or being on treatment for a common mental health condition at baseline is consistent with the results of a range of studies [14, 18, 21–24, 37, 38, 40, 41]. We also found that women reported loneliness more commonly than men, something which is also consistent with the findings of others [3, 14, 39]. It may be that the sex differences reflect differences in social relationships between men and women and/or differences in health habits and outcomes [42, 43]. However, it is equally possible that men tend to be more hesitant than women to report loneliness, due to culturally related sex biases [44, 45]. Loneliness has most often been studied amongst older people, making evidence on the relationship between job characteristics and risk of loneliness sparse. However, unemployment has been found to be associated with the risk of loneliness amongst more than 1000 adults with a mean (SD) age of 45.1 (15–44) years [8] and more than 600 adults aged 50–65 years [7]. Although both studies were cross-sectional in design, they would seem to point to a protective effect of being in employment against the risk of loneliness. In this analysis, however, we did not find convincing evidence of an increased odds of loneliness among people who exited the workforce compared with those who remained in work throughout. Of course, not all jobs (or employers) are the same. Our analysis additionally explored the effects of perceived psychosocial work characteristics, representing the individual’s subjective view of their job, for those still working at the end of follow-up, and found that some of the negative psychosocial factors were associated with loneliness 2 years later.

Our findings also indicated that permanent job exit (mostly because of retirement) did not predict loneliness, in contrast to our original hypothesis that loneliness at older ages was partly due to loss of social relationships at work. It is possible that the social contact at work in poorer quality jobs is insufficient or ineffective and therefore contributes to the associations with loneliness that we demonstrated. However, if this is the case, it is interesting that we did not find an association with loneliness amongst people reporting poor support at work from supervisors/colleagues. It is possible that a 2-year interval is too short a time period for the effects of loss of social contact at work to become noticeable. Interestingly, the stability of loneliness over time was recently described in a lifecourse meta-analysis of longitudinal studies performed by Mund and colleagues [46]. They reported that mean-level loneliness showed a U-shaped distribution, decreasing throughout childhood to a level which is then relatively stable throughout adulthood until the oldest of old ages. Their data did not appear to show a large effect of retirement, but we will be better placed to investigate these effects over longer periods of time in the HEAF study in the future.

Other negative health impacts of adverse psychosocial work factors have been reported, including sleep disorders [14, 21, 22], poor mental health and depressive symptoms [40, 41], poor SRH and well-being in older workers [42]. The evidence suggests a close relationship between mental health, depression and the risk of loneliness, and that exposure to adverse work stressors, which can negatively impact mental health, may also affect the risk of loneliness. Our finding is important as there is potential for work stressors to be identified, managed and reduced by developing better quality, more rewarding jobs that retain older workers in health and comfort.

Our study has a number of strengths. HEAF is a large contemporary population-based cohort of older working-age individuals distributed all over England, and representing all deciles of deprivation. Almost everyone in Britain registers with a general practice for healthcare that is free at the point of delivery, so patient lists from general practices offer a comprehensive sampling frame. The cohort includes people doing a wide range of different jobs and living in a wide variety of social circumstances. The longitudinal design of our study allows for the associations reported to be regarded as potentially causal. A further strength of this study is the wide range of available information about the job environment and participants’ perception of such environment.

Our results do, however, need to be considered alongside some limitations. At baseline, the overall response rate was fairly low (21%), and we have previously shown that HEAF responders tended to be somewhat older, more affluent and more likely female than non-responders [30]. Even though this might have affected the generalisability of our results, the HEAF study has an excellent retention rate (> 90%) so that internal comparisons within the cohort have a high rate of validity. Another potentially-important limitation to the current research is the use of a single self-labelling item to assess the outcome loneliness. Data collection for the HEAF study has, to date, been undertaken by annual postal questionnaires. Our questionnaires are designed to collect a wide range of information about exposures and outcomes to answer the most clinically important and relevant research questions. Unfortunately, if all measures were obtained using their validated tools, the questionnaires would be bulky and burdensome and we would expect much lower rates of retention than those currently achieved. Therefore, we have needed to compromise by using some measures in their shortened form, including using the CES-D question for loneliness. However, we chose to do this in the light of convincing evidence that this measure has been shown to have moderately strong correlation with the validated UCLA loneliness scale [32] and that there is face validity for doing so, in that other authors have used the same measure [45, 47–49]. .

Since 1984, when the ILO/WHO Committee published their definition of psychosocial characteristics [26], our understanding of these has grown and some complex models have been established including the demand-control-support and effort-reward imbalance models but also new factors including e.g. bullying and discrimination. As reported above, some exposure information in HEAF has needed to be collected through single-item questions derived from existing validated tools. We acknowledge that this is the case for the psychosocial factors used in the current analyses and that this is a weakness of the current study. However, single-item measures do have a role in studies such as this [50] and we note that we have been able to add some of the most important validated tools into future rounds of the questionnaire so that we can revisit these analyses in future HEAF publications. Finally, the psychosocial work factors used in the current analyses were those reported at baseline. It was therefore possible that the psychosocial characteristics changed if the participant had moved job: we found that 26% of participants had changed their job between baseline and follow-up. Therefore, we repeated our analyses only including those who had held the same jobs throughout and obtained broadly similar results.

It is noteworthy that the number of participants who exited paid work during follow-up was relatively small (7.5%) and these low numbers may have reduced our chances of finding an effect of exit on loneliness, particularly if the risk of loneliness increases with years away from work. This relatively low attrition also prevented us exploring our research question about the effect of voluntary work exit (retirement) on loneliness separately from those with forced job exit caused by unemployment, which will be reported in a future investigation. Another limitation of our study is the fact that a 2-year follow-up window might be rather narrow to fully capture permanent exits from the workforce.

## Conclusions

In this study we found that adverse psychosocial work characteristics amongst workers aged 50–64 years increased the odds of loneliness 2 years later. In particular, often lying awake worrying about work, rarely having a choice in the workplace and not coping with the physical demands of the job were important risk factors. We also found that such associations were similar when we restricted the analyses to older workers still in work at the end of follow-up, as opposed to those that had exited the workforce. These results suggest that working to older ages alone will not reduce the risk of loneliness among older people, but that there is the potential to reduce the risk of loneliness by improving job quality and addressing the negative effects of perceived workplace stressors.

## Supplementary Information

**Additional file 1: Appendix 1** DAG.pdf: Directed Acyclic Graph for selection of confounders

**Additional file 2: Appendix 2** Cross-sectional baseline characteristics by loneliness status.doc: Baseline characteristics of participants according to loneliness status

## Data Availability

The datasets used and/or analysed during the current study are available on reasonable request from the “MRC Versus Arthritis Centre for Musculoskeletal Health and Work” by contacting. Dr. Holly Syddall: hes@mrc.soton.ac.uk or. Dr. Catherine Linaker: chl@mrc.soton.ac.uk The HEAF study is part of the Cohort and Longitudinal Studies Enhancement Resources (CLOSER), the home of longitudinal research, bringing together longitudinal studies: https://www.closer.ac.uk/. Data and questionnaires from HEAF can be searched and browsed using the CLOSER Discovery search engine: https://discovery.closer.ac.uk/.
