# Supplementary figures and images for: Older working adults in the HEAF study are more likely to report loneliness after two years of follow-up if they have negative perceptions of their work quality
Source: BMC Public Health. 2021 Mar 23;21:574. doi: 10.1186/s12889-021-10610-5 (PMC7988922; doi:10.1186/s12889-021-10610-5)

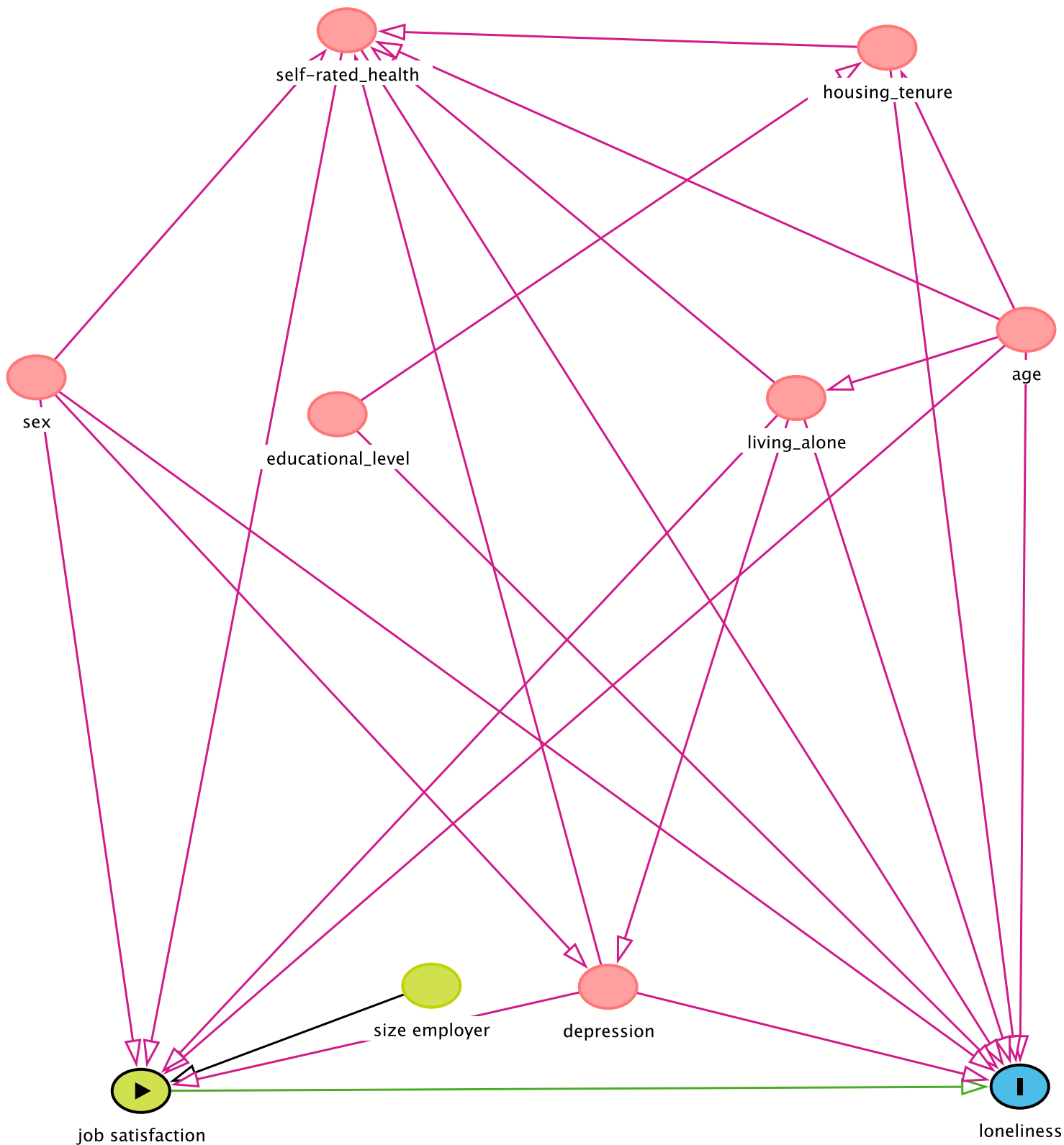

Supplement: Supplementary file 1 — Additional file 1: Appendix 1 DAG.pdf: Directed Acyclic Graph for selection of confounders [file 12889_2021_10610_MOESM1_ESM.pdf]
