# Supplementary material for: Older working adults in the HEAF study are more likely to report loneliness after two years of follow-up if they have negative perceptions of their work quality
Source: BMC Public Health. 2021 Mar 23;21:574. doi: 10.1186/s12889-021-10610-5 (PMC7988922; doi:10.1186/s12889-021-10610-5)
Supplement: Supplementary file 2 — Additional file 2: Appendix 2 Cross-sectional baseline characteristics by loneliness status.doc: Baseline characteristics of participants according to loneliness status [file 12889_2021_10610_MOESM2_ESM.docx]

**OLDER WORKING ADULTS IN THE HEAF STUDY ARE MORE LIKELY TO REPORT LONELINESS AFTER TWO YEARS OF FOLLOW-UP IF THEY HAVE NEGATIVE PERCEPTIONS OF THEIR WORK QUALITY**

**Running title: Work, psychosocial work factors and loneliness**

Dr Gregorio Bevilacqua^1^

Ms Stefania D’Angelo^1^

Dr Georgia Ntani^1,2^

Dr Holly Emma Syddall^1^

Dr Elizabeth Clare Harris^1,2^

Dr Cathy Linaker^1,2^

Mr Martin Stevens^1,2^

Prof Cyrus Cooper^1,2^

Prof Karen Walker-Bone^1,2^

^1^ MRC Lifecourse Epidemiology Unit, University of Southampton,

^2^ MRC Versus Arthritis Centre for Musculoskeletal Health and Work, MRC Lifecourse Epidemiology Unit, University of Southampton

Correspondence and reprint requests to: Professor Karen Walker-Bone, MRC Lifecourse Epidemiology Unit, University of Southampton, Southampton General Hospital, Southampton, SO16 6YD, UK. email: [kwb@mrc.soton.ac.uk](mailto:kwb@mrc.soton.ac.uk)

**Appendix Table 2: Cross-sectional baseline characteristics by loneliness status***

| **Psychosocial work factors, n (%)** | **Overall**  **(n=5,473)** | **Rarely/some of the time lonely (n=4,896)** | **Occasionally/most of the time lonely (n=577 )** |
| --- | --- | --- | --- |
|  |  |  |  |
| Rarely having choice at work | 1069 (19.9%) | 910 (18.9%) | 159 (28.3%) |
| Lack of support | 607 (11.3%) | 520 (10.8%) | 87 (15.4%) |
| Often worrying about work | 699 (12.9%) | 542 (11.2%) | 157 (27.6%) |
| Rarely feeling of achievement | 395 (7.3%) | 299 (6.2%) | 96 (16.9%) |
| Rarely feeling of appreciation | 557 (10.3%) | 442 (9.2%) | 115 (20.3%) |
| Job dissatisfaction | 363 (6.7%) | 264 (5.5%) | 99 (17.4%) |
| Not coping with physical demand | 1589 (29.5) | 1326 (27.5%) | 263 (46.3%) |
| Not coping with mental demand | 1711 (31.7) | 1413 (29.3%) | 298 (52.7%) |

*All p-values < 0.001 (not shown)
